# Anaphylaxis as a Manifestation of Horse Allergy

**DOI:** 10.1097/WOX.0b013e3181b2fe51

**Published:** 2009-08-15

**Authors:** Radoslaw Gawlik, Tomas Pitsch, Lawrence DuBuske

**Affiliations:** 1 Department and Clinic of Internal and Allergic Diseases and Clinical Immunology, Silesian University School of Medicine, Zabrze, Poland; 2 Immunology Research Institute of New England, Harvard, MA

**Keywords:** anaphylaxis, allergy, horse

## Abstract

Allergic disease induced by animal exposure is a common phenomenon worldwide. Whereas cat and dog dander exposure are well recognized as causative of allergic rhinitis, allergic asthma, and contact urticaria, horse allergy can present with anaphylaxis. Horse allergy is induced by exposure to the major horse allergens Equ 1 through 5. The severity of the symptoms may be related to the level of exposure. Greatest risk of anaphylaxis occurs in those sensitized patients who have large amounts of animal allergen exposure, such as when in a barn, or when an animal bite occurs exposing sensitized persons to large quantities of the animal allergen that resides in the saliva. Horse allergy may be successfully treated with allergen specific immunotherapy.

## Introduction

Allergic diseases are among the most common afflictions worldwide. Allergy to pets, such as cats and dogs, causes significant morbidity in children. The most commonly reported manifestations of pet allergy include asthma, contact urticaria, angioedema, and contact dermatitis. Horses are arguably the most important animal in history to be domesticated. For centuries, horses have been used as a means of transportation, pleasure, work, and even war. Today horses are rarely used for work in developed countries but are widely owned for recreational activities. Horses have been recognized as an important source of allergens. Horse allergy occurs in people who regularly work with horses, either professionally or for recreational purposes, and in people indirectly exposed to horses through allergens on riding clothes. Despite the presence of horses in a variety of recreational, sporting, and work environments, there are only a few reports of anaphylactic episodes resulting from horse allergy [1].

## Case Report

This is a case of a six-year-old girl, with a history of atopic dermatitis and respiratory allergies, who was admitted to the hospital in January, 3 days after an episode of anaphylaxis. The reaction occurred shortly after a 10-minute ride in a snow sleigh pulled by a horse. Her symptoms included sneezing, rhinitis, erythematous skin rash, periorbital angioedema, and lip swelling. Her teacher also reported that the child had difficulty breathing, and wheezing. The girl was transferred to the emergency ward. Physical examination demonstrated tachypnea, a respiratory rate of 32/min, tachycardia-pulse of 110/min, hypotension-blood pressure of 80/50 mm Hg, and an axillary temperature of 36.3°C. Lung auscultation revealed wheezes and crackles throughout all lung fields. The presence of respiratory, dermal, and cardiovascular symptoms were consistent with a Muller Class 3 systemic allergic reaction. In the emergency department, she received salbutamol MDI 100 μg/puff, intramuscular clemastine (1 mg), intravenous hydrocortisone (400 mg), oral prednisone (20 mg), and oxygen administration and rapid clinical improvement observed.

After admission to the hospital her physical examination, spirometry, and chest radiographs were normal. She was hospitalized to monitor her allergic reaction and perform diagnostic procedures. TSkin prick tests with all inhalant allergens (Allergopharma, Germany) were negative, as was the histamine positive control. The results of the skin prick tests conducted 4 days after treatment of anaphylaxis were likely influenced by the use of several medications used as part of the anaphylaxis treatment. Specific IgE (DPC, Biermann, Germany) levels were markedly elevated against horse dander (> 100 kU/l), cat and dog dander 2.43 and 5.95 kU/l, respectively, and mugwort, birch, alder, and hazelnut tree pollens, 0.15 kU/l. Total serum IgE (DPC, Biermann, Germany) was 248 kU/l. Spirometry throughout the hospitalization remained normal.

Two weeks after admission to the hospital, skin prick tests with common inhalant and food allergens were performed again. Skin prick tests, except to horse, cat and dog epithelium, and milk, were negative. The wheal diameters were as follows: 14 mm for horse, 5 mm for cat, 4 mm for dog, 4 mm milk, and 4 mm for histamine HCl 0.1%.

As the anaphylaxis occurred outdoor in winter, an ice cube challenge to the forearm was performed and was negative. Avoidance of the exposure to furred animals, especially horse, was advised. She tolerates contact with cats and dogs very well. When she was an infant she did not tolerate milk and was treated because of atopic dermatitis. The symptoms disappeared at 3 years of age.

Two months later, exposure to horse allergen accidentally occurred when the girl very briefly visited an empty stable. That incident elicited a vigorous early asthmatic re-sponse with bronchospasm. Spirometry performed when she presented to the clinic after 1 hour showed a 25% fall in FEV1 (Jaeger, Germany). Treatment with inhalation of *β*2 agonists and oxygen resolved the symptoms in 1 hour. The patient was instructed to strictly avoid horses and stables.

### Mammalian Allergens in Allergic Diseases

Allergic diseases including asthma and anaphylaxis are all connected with exposure to different allergens. Among them, animal allergens play an increasing role. Animal allergens are common causes of both acute and chronic allergic diseases. Among the animal allergens that cause allergic diseases the most important are those derived from cats, dogs, rats, mice, cows, and horses. These mammals secrete and excrete allergens into the environment. Allergic sensitization may occur at home or in the workplace, directly or indirectly, with the latter because of transportation of allergens on clothing such as riding clothes as has been anecdotally noted in the past [2].

Animal allergens are large enough to act as complete antigens and induce production of allergen specific immunoglobulin IgE and IgG. The structural basis may play a role in modulating the immune response in favor of T_H_2 dominance.

Although much is known about allergic reactions caused by allergens such as cat and dog dander, and allergies to rats and mice are common among laboratory workers, horse allergens as a potential cause of severe allergic reaction are often forgotten and underestimated.

### Animal Allergens in the Environment

Cats and dogs are the animals most commonly causing allergies affecting the general population. Sensitivity to cat and dog allergens has been shown to occur in 22 to 67% of asthmatic patients. A recent study measuring the prevalence of allergen sensitization among 6500 7-year-old British children showed the after allergen sensitization pattern: grass pollens allergy in 8.5%, *D. pteronyssinus *allergy in 7.8%, cat in 4.9%, *D. farinae *allergy in 3.6%, dog allergy in 2.7%, horse allergy in 1.4%, and rabbit allergy in 1.4% of the study population [3]. These results demonstrate that horse dander is still a major allergen causing sensitazation in children despite lack of regular exposure.

Cat and dog allergen concentrations are highest in homes where these animals live. However, these allergens can also be found in homes that have never had pets. Passive transfer of proteins from one place to another is the likely cause of the phenomenon [4]. The allergen levels in homes without dogs or cats are usually lower than in homes that have animals, but the concentrations are often great enough to cause sensitization.

Several studies in Europe have indicated that early exposure to farm animals has a protective effect against both allergy and asthma [5-7]. Children growing on farms rarely suffer on allergic diseases [5-7]. The presence of animals in the home environment in the first year of life may be protective insofar as development of immune responses involving Immunoglobulin(Ig)G directed toward animal dander [8,9].

The greatest concentrations of other mammal allergens are observed in places where these animals are present. However, no clear association was observed assessing allergy and exposure to different animal species on a farm [10]. High levels of mouse and rat allergens have been noted to be present in laboratories, inducing allergic sensitization and sometime disabling allergic disease symptoms in laboratory workers. A clear dose-response of symptoms was demonstrated in laboratory workers with rat allergy [11]. Subjects who have regular contact with laboratory animals often have sensitivity to these animals. A very large epidemiological study involving more than 5000 laboratory animal workers in Japan demonstrated symptoms in 26% of workers exposed to mouse, 25% exposed to rats, 315 exposed to guinea pigs, in 30% exposed to rabbits, in 26% exposed to hamsters, in 30% exposed to cats, in 25% exposed to dogs and in 24% exposed to monkeys [12].

### Horse Allergens

Horses are considered to be one of the most important and significant sources of mammalian allergens. Allergen extracts from horse dander, hair, and skin scraping are the usual sources of horse inhalant. Their protein content differs whereas the carbohydrate content is of the same order. Many efforts have been made to enhance the biochemical and immunologic characterization of the various horse allergen containing extracts. The first allergic reactions associated with horse allergens were described in the 1950s after injection of therapeutic horse serum. Horse serum albumin was reported as the first known horse allergen [13]. In the 1970s, 3 dander derived allergens, designated Ag 6, Ag 9, and Ag 11, were described [14]. Subjects with allergy to horse possess specific IgE directed against these 3 antigens in 54, 90, and 100% of cases, respectively. Horse allergens are proteins, mainly acid, with an apparent molecular mass range between 10 kDa and 75 kDa [14].

Only 4 horse allergens have been purified to homogeneity and characterized. In the late 1990s, 2 important horse allergens, Equ c1 and Equ c2, members of the lipocalin superfamily were characterized.

Equ c1, a major horse allergen, hair dandruff protein, the most important from a clinical perspective, is an acidic glycoprotein (molecular weight 25 kD) synthesized in the liver and in the sublingual and submaxillary salivary glands [13]. Antigens Equ c2 (molecular weight 17 kD) and Equ c3-*Ag9 *(molecular weight 16 kD) were isolated from horse sweat [15]. Equ c3 is a horse serum albumin member of important crossreactive protein family serum albumins containing, for example, allergens of cat (Fel d2), dog (Can d3), and cattle (Bod d6) [16,17]. The same investigators identified Equ c4 (molecular weight 16.7 kDa) that shares a significant sequence homology with the rat submandibular gland protein A [10]. In the course of purification of Equ c4, a new allergen from horse dander named Equ c5 (molecular weight 18.7 kDa), has been identified and characterized [16].

The amino acid sequence of horse allergens shows about 50% homology with rodent urinary proteins, suggesting that Equ c1 belongs to the lipocalin family. Equ c2 is a protein (20 kD) with about 50% similarity to Bos d2, a major cow dander allergen. A high degree of sequence similarity (60-71%) was also found with a number of proteins belonging to the lipocalin family. This family includes most mammalian allergens [18]. Lipocalins are a large group of proteins that bind and transport small hydrophobic molecules. Cockroach (Bla g4), rat (Rat n1), mouse (Mus m1), and milk (beta-lactoglobulin) allergens belong to this family of proteins. The allergens of mammalian origin usually present in hair dandruff, such as Fel d1, Can f1, Can f 2, Bov d1, Mus m1, Rat n1, and Rat n2, are also rather acidic. The allergenicity of lipocalins is a consequence of molecular mimicry between endogeneous lipocalins and exogenous lipocalin allergens at the T-cell level. Horse serum albumin cross-reacts with dog and cat serum albumin [16]. Study on horse allergen composition has revealed some interspecies differ-ences. Although the molecular structures of the horse allergens are well known, the immunologic cellular responses to those allergens are not yet clear. There was a popular opinion that Bashkir horses were nonallergic and this breed of horses was recommended to allergic patients. Study on allergenic content of different horse breeds unfortunately failed to confirm this viewpoint, and confirmed presence of the same allergens in other breeds [19]. However, authors conclude that there is pronounced variation in the allergenic composition of dander extracts among individual horses.

### Environmental Prevalence of Horse Allergens

The dispersion of allergens from furred animals to pet-free public places is likely to occur by deposition from people who have been in direct or indirect contact with pets, and high levels of such allergens seem to accumulate in a short period of time [20]. Horses are able to generate large amounts of airborne allergens. Elevated levels of horse allergen were found outdoors often in the close vicinity of stables. Levels of airborne horse allergen were more than 500-fold higher in the stable than just outside the stable and more than 3000-fold greater than at a nearby residence located only 12 meters from the stable [21]. Horse allergens in air samples collected 40 meters from the stable were undetectable [21].

The frequency of horse allergy depends on its presence in environment and equestrian tradition. Sensitization to horse epithelium in Spain in the region Huelva was found in 12.3% of all patients attending an Outpatient Clinic and was there the fifth most prevalent allergen [22].

A study by Sovalainen [23] demonstrated the prevalence of allergies to large furred farm animals and documented respiratory symptoms among groups of people who are highly exposed to these allergens. Turkish authors revealed that 12.8% of grooms working in the hippodrome in Istanbul had sensitization to horse hair, proving that occupational exposure to horses increases the respiratory and other allergic symptoms [24].

A week of persistent deterioration of asthma occurring many hours after exposure to horse suggests that horse allergens may provoke late phase allergic symptoms. The case reported by Roberts and Lack [2] described an episode of anaphylaxis in an 8-year-old boy after riding a pony. This case supports the concept that horse dander allergen may cause life-threatening anaphylaxis. It is likely that children are especially susceptible to horse allergens. Our case demonstrates that there might be an increased risk of anaphylaxis after contact with large amounts of horse allergens in children who have had long contact with horses, so that exposure occurs to great concentrations of allergens over time, and as a result, induces symptoms of anaphylaxis.

### Symptoms of Horse Allergy

Horse allergy occurs among people who regularly handle with horses, either professionally or for recreational purposes, and is mainly characterized by rhinitis, conjunctivitis, asthma, and occasionally by urticaria. Symptoms are highly correlated with the levels of allergen exposure.

A study by Lelong of 56 children and adolescents allergic to horses, noted that allergic disease presented with ocular symptoms in 36 patients, asthmatic in 30 patients, and rhinitis in 24 patients [25]. A case of contact urticaria triggered by horse saliva has been described [26]. Unusual events could induce anaphylactic reactions in horse sensitized people. Guida reported a systemic anaphylactic reaction in a horse allergic patient who had previously only had allergic rhinitis when exposed to horses after a horse bite. This anaphylactic reaction was probably because of lipocalin sensitization [27].

### Diagnosis of Horse Allergy

Because avoidance of allergens is critical in the management of allergy, especially when it is as simple as in the case of a horse allergy, proper diagnosis is crucial for appropriate management. Skin prick tests, being both simple and inexpensive, are a useful investigative tool. In combination with a thorough history and clinical examination, skin prick tests may be sufficient to diagnose allergy to horse. As an example, all the children in the Lelong study had very positive skin prick tests and allergen specific IgE [25] but no patient was mono-sensitized to horse.

In our case, this kind of allergy was also confirmed by elevated serum specific IgE level against horse epidermis. High levels of serum IgE antibodies against cat and dog dander were also observed, but the girl did not report any symptoms associated with exposure to these animals. The observed reaction could have occurred in response to contact with allergens contained in the animal dander, secretions, and excretions.

### Treatment of Animal Allergy

Avoidance of allergens is critical in the management of horse or other animal allergy. Cat dander may persist for years even after removing of cats from the home environment. The inhaled cat allergen may be effectively removed by thorough cleaning of home and may be below the threshold level that induces clinical responses. HEPA filtration of the ambient home air may also help remove animal allergens.

The most appropriate treatment is to remove the animal from the home of the person who is sensitive. Removing an animal from the home has confirmed value in secondary prevention. It is necessary to remember that the allergen levels decrease slowly over several months after pet removal. In families that are not willing to eliminate their pets from their homes other methods are to be considered including: washing pets intensively, the usage of air cleaners, the removal of allergen reservoirs such as carpets, and the usage of mattress and pillow covers [28]. All of these measures are useful in reducing exposure. Important advice to give patients is to keep the pets in one restricted area of the home, especially out of patient's bedroom. However, a high proportion of patients are either reluctant or completely unwilling to remove a household pet.

Prophylactic measures in farm animal allergy are simple and require avoidance of places where animal live. The patient should be instructed to strictly avoid horses, barns, and stables.

As the allergy to the animal allergens symptoms include the anaphylactic shock appropriate, rescue treatment including injectable epinephrine, oral antihistamines, and inhaled *β*2 agonists should be prescribed for patients with solely respiratory symptoms because of animal allergy. Patients with a previous severe anaphylactic systemic reaction should carry a preloaded epinephrine device and should be educated when and how to use this device. Allergen immunotherapy may be the treatment of choice in many cases of animal allergy, especially when the avoidance of allergen is not possible [29].

The clinical uncontrolled study of Fernandez-Tavora designed to desensitize 24 horse allergic patients showed high safety and efficacy of horse allergen specific immunotherapy [22]. All of these patients presented with asthma and rhinoconjunctivitis symptoms with or without cutaneous symptoms. A purified extract from ALK-Abello was used for immunotherapy in a cluster schedule (87%) or a conventional (13%) schedule and then maintenance doses were administered from 2.1 up to 653 months (median 7.4 month). Five adverse reactions were observed in 4 patients. SIT was not withdrawn in any one case. Efficacy of the immunotherapy was very good, 95% of patients and investigators opinions were excellent (65%) or good (30%). Most of the patients were highly satisfied and could continue their hobbies or professional activities. With horse-riding exposure after SIT significantly, patients who had ASIT were noted to have reduction in the after symptoms: 100% reduction in conjunctivitis symptoms, 93% reduction in rhinitis symptoms, 90% reduction in asthma symptoms, and an 87% reduction in cutaneous symptoms [22].

Symptomatic treatment of horse induced respiratory allergy should also be considered, including, leukotrienes antagonists, systemic and topical antihistamines, or topical or even systemic glucocorticosteroids.

## Discussion

In several children, the first manifestations of horse allergy may occur at the time of the first known contact with a horse or pony. This case of a girl accidentally exposed to horse allergens shows that horse allergy may be an important clinical problem with dramatic sudden presentation, especially in children. It should be differentiated from other types of reactions including cold urticaria, exercise induced anaphylaxis, or food anaphylaxis. Roberts and Lack, when reporting 3 cases of a horse allergy, noted that horse dander is often not taken into consideration as a cause of an allergic disease, including asthma, especially in an urban environment [2]. Describing a case of a 9-year-old boy suffering from asthma who was undergoing a 50% decline in his peak expiratory flow every Friday after a contact with his sister's riding gear; they suggested that horse allergens can be carried on clothing. Thus, horse allergens may exist in the indoor environment and may cause an allergic reaction in patients who have not had any contact with a horse. The possibility of crossreaction between patients allergic to horse albumin (Equ c3) and dog, cat, or guinea pig albumin should be stressed [16,17] Horse allergy may cause varying clinical symptoms, such as urticaria, angioedema, rhinitis, or respiratory distress, and the onset of symptoms may be delayed. Indirect exposure to horse dander carried on clothing into the indoor environment should be taken into account. Furthermore, horse allergens may be present in an urban environment, not only in rural environment. Patients often overlook and mistake allergy to horses, for allergy to pollens or molds. In disabled children, allergy to horses must be considered when clinical signs of allergy occur during therapeutic riding sessions.

## Conclusion

Unexpected exposure to uncommon allergens should be sought as a cause of unexplained anaphylaxis. Horse dander as an allergen is now often overlooked as a possible cause of anaphylaxis. Whenever anaphylaxis has no clear causation, investigation should consider mammalian allergen exposure as a possible cause. Horse dander as an allergen may be relevant to both the rural and the farm environments as both direct and indirect exposure to this potent allergen may lead to severe life threatening allergic responses.

## References

[B1] Chapman MD Wood RA The role and remediation of animal allergens in allergic diseases J Allergy Clin Immunol 2001 2 414 421 10.1067/mai.2001.113672 11242602

[B2] Roberts G Lack G Horse allergy in children BMJ 2000 2 286 287 10.1136/bmj.321.7256.286 10915137 PMC1118279

[B3] Roberts G Peckitt C Northstone K Relationship between aeroallergen and food allergen sensitization in childhood Clin Exp Allergy 2005 2 933 940 10.1111/j.1365-2222.2005.02280.x 16008681

[B4] Almqvist C Larrson PH Egmar A-C Hedren M Malmberg P Wickman M School as a risk environment for children allergic to cats and a site for transfer of cat allergen to homes J Allergy Clin Immunol 1999 2 1012 1017 10.1016/S0091-6749(99)70172-7 10359879

[B5] Riedler J Eder W Oberfeld G Schreuer M Austrian children living on a farm have less hay fever, asthma and allergic sensitisation Clin Exp Allergy 2000 2 194 200 10.1046/j.1365-2222.2000.00799.x 10651771

[B6] Kilpelainen M Terho EO Helenius H Koskenvuo M Farm environment in childhood prevents development of allergies Clin Exp Allergy 2000 2 201 208 10.1046/j.1365-2222.2000.00800.x 10651772

[B7] Ernst P Cormier Y Relative scarcity of asthma and atopy among rural adolescents raised on a farm Am J Respir Crit Care Med 2000 2 1563 1566 10.1164/ajrccm.161.5.9908119 10806155

[B8] Ownby DR Johnson CC Peterson EL Exposure to dogs and cats in the first year of life and risk of allergic sensitization at 6 to 7 years of age JAMA 2002 2 963 972 10.1001/jama.288.8.963 12190366

[B9] Platts-Mills T Vaughan J Squillace S Sensitisation, asthma, and a modified Th2 response in children exposed to cat allergen: a population based cross-sectional study Lancet 2001 2 752 756 10.1016/S0140-6736(00)04168-4 11253969

[B10] Goubran Botros H Poncet P Rabillon J Fontaine T Laval JM David B Biochemical characterization and surfactant properties of horse allergens Eur J Biochem 2001 2 3126 3136 10.1046/j.1432-1327.2001.02217.x 11358533

[B11] Egglestone PA Ansari AA Adkinson NF Wood RA Environmental challenge studies laboratory animal allergy Am J Respir Crit Care Med 1995 2 640 646 10.1164/ajrccm/151.3_Pt_1.640 7881650

[B12] Aoyama K Ueda A Manda F Matsushita T Ueda T Yamauchi C Allergy to laboratory animals: an epidemiologic study Br J Ind Med 1992 2 41 47 1733454 10.1136/oem.49.1.41 PMC1039233

[B13] Gregoire C Rosinski-Chupin I Rabillon J cDNA cloning and sequencing reveal the major horse allergen Equ c 1 to be a glycoprotein member of lipocalin superfamily J Biol Chem 1996 2 32951 32959 10.1074/jbc.271.51.32951 8955138

[B14] Lowenstein H Markussen B Weeke B Isolation and partial characterization of three major allergens of horse hair and dandruff Int Arch Allergy Appl Immunol 1976 2 4867 10.1159/000231578 1279018

[B15] Goubran Botros H Rabillon J Gregoire C David B Dandeu JP J Chromatogr B Thiophilic adsorption chromatography: purification of Equ c2 and Equ c3, two horse allergens from horse sweat Biomed Sci Appl 1998 2 57 65 10.1016/S0378-4347(98)00130-3 9686871

[B16] Goubran Botros H Gregoire C Rabillon J David B Dandeu JP Cross-antigenicity of horse serum albumins: study of tree short peptides with significant inhibitory activity towards specific human IgE and IgG antibodies Immunology 1996 2 340 347 10.1046/j.1365-2567.1996.d01-669.x 8774348 PMC1456354

[B17] Spitzauer S Pandjaitan B Soregi G Mühl S Ebner C IgE cross-reactivities against albumins in patients allergic to animals J Allergy Clin Immunol 1995 2 951 959 10.1016/S0091-6749(95)70233-4 8543754

[B18] Virtanen T Zeiler T Mantyjarvi R Important animal allergens are lipocalin proteins: why are they allergenic? Int Arch Allergy Immunol 1999 2 247 258 10.1159/000024277 10640908

[B19] Felix K Ferrandiz R Einarsson R Dreborg S Allergens of horse dander: comparison among breeds and individual animals by immuno-blotting J Allergy Clin Immunol 1996 2 169 171 10.1016/S0091-6749(96)70239-7 8765831

[B20] Egmar AC Almqvist C Emenius G Lilja G Wickman M Deposition of cat (Fel d1), dog (Can f1), and horse allergen over time in public environments a model of dispersion Allergy 1998 2 957 961 10.1111/j.1398-9995.1998.tb03796.x 9821475

[B21] Emenius G Larsson PH Wickman M Härfast B Dispersion of horse allergen in the ambient air, detected with sandwich ELISA Allergy 2001 2 771 774 10.1034/j.1398-9995.2001.056008771.x 11488672

[B22] Fernandez-Tavora L Rico P Martin S Clinical experience with specific immunotherapy to horse dander J Investig Allergol Clin Immunol 2002 2 29 33 12109529

[B23] Savalainen J IgE response to fur animal allergens and domestic animal allergens in fur farmers and fur garment workers Clin Exp Allergy 1997 2 5 12 9179423

[B24] Tutluoglu B Atis S Anakkaya AN Altug E Tosun GA Yaman M Sensitization to horse hair, symptoms and lung function in grooms Clin Exp Allergy 2002 2 1170 1173 10.1046/j.1365-2745.2002.01439.x 12190654

[B25] Lelong M Castelain MC Bras C An outbreak of allergy to horses in children. A review of 56 recent cases Pediatrie 1992 2 55 58 1337779

[B26] van der Mark S Contact urticaria from horse saliva Contact Dermatitis 1983 2 145 10.1111/j.1600-0536.1983.tb04323.x 6851523

[B27] Guida G Nebiolo F Heffler E Bergia R Rolla G Anaphylaxis after horse bite Allergy 2005 2 1088 1089 10.1111/j.1398-9995.2005.00837.x 15969694

[B28] van der Heide S van Aalderen WM Kauffman HF Dubois AE de Monchy JG Clinical effects of air cleaners in homes of asthmatic children sensitized to pet allergens J Allergy Clin Immunol 1999 2 447 451 10.1016/S0091-6749(99)70391-X 10452769

[B29] Nelson HS Advances in upper airway diseases and allergen immunotherapy J Allergy Clin Immunol 2005 2 676 684 10.1016/j.jaci.2005.01.037 15805984

